# Methicillin-resistant Staphylococcus aureus outbreak in a Dutch equine referral clinic

**DOI:** 10.1099/jmm.0.001873

**Published:** 2024-08-29

**Authors:** Marleen M. Kannekens-Jager, Birgitta Duim, Linda van der Graaf-van Bloois, Aldert L. Zomer, Marian J. Broekhuizen-Stins, Maarten Boswinkel, Jaap A. Wagenaar, Els M. Broens

**Affiliations:** 1 Division of Infectious Diseases and Immunology, Department of Biomolecular Health Sciences, Faculty of Veterinary Medicine, Utrecht University, Utrecht, Netherlands; 2 Animal Hospital De Visdonk, Roosendaal, Netherlands

**Keywords:** antimicrobial resistance, equine clinic, MRSA, MRSA outbreak, whole-genome sequencing

## Abstract

In 2020 and 2022, nine cases of surgical site infections with a methicillin-resistant *Staphylococcus aureus* (MRSA) were diagnosed in horses in an equine referral clinic. Sixteen isolates (horses, *n*=9; environment, *n*=3; and staff members, *n*=4) were analysed retrospectively using Nanopore whole-genome sequencing to investigate the relatedness of two suspected MRSA outbreaks (2020 and 2022). The MRSA isolates belonged to ST398 and ST612. ST398 genomes from 2020 and 2022 formed three phylogenetic clusters. The first ST398 cluster from 2020 consisted of isolates from five horses and one staff member, and we suspected within clinic transmission. The second cluster of ST398 isolates from 2022 originated from two horses and two staff members but showed higher single nucleotide polymorphism (SNP) distances. One ST398 isolate from an individual staff member was not related to the other two clusters. The ST612 isolates were isolated in 2022 from two horses and three environmental samples and showed very low SNP distances (<7 SNPs), indicating the transmission of MRSA ST612 in this clinic in 2022. Molecular characterization revealed an abundant set of virulence genes and plasmids in the ST612 isolates in comparison to ST398 isolates. Phenotypic antimicrobial susceptibility showed that differences between the two sequence types were consistent with the genetic characteristics. MRSA ST612 has not been reported in Europe before, but it is a dominant clone in African hospitals and has been described in horses and people working with horses in Australia, indicating the importance of surveillance.

## Introduction

Methicillin-resistant *Staphylococcus aureus* (MRSA) is one of the ESKAPE organisms [1] and is responsible for both hospital- and community-associated infections worldwide and therefore a major public health concern. Most of the reported equine hospital outbreaks in Europe are associated with MRSA clonal complex (CC) 398 [2], but in other parts of the world, other CCs and sequence types (STs) have been described [3,5]. MRSA carriage in horses poses a zoonotic risk and causes a risk for infection and transmission between horses and between horses and staff members in equine hospitals. The prevalence of MRSA in healthy horses ranges from 0 to 6% [3]. In hospitalized horses, the MRSA prevalence can increase up to 40–50%, and several outbreaks in equine hospitals have been described [3,6]. When MRSA outbreaks in veterinary (equine) hospitals occur, often, additional investigation is performed to screen for MRSA colonization in staff members (veterinarians and caretakers) in these hospital settings. The investigation of MRSA colonization in veterinarians working with horses in Australia showed a prevalence of 11.8% [7]. In Germany, the prevalence of nasal MRSA colonization in veterinary personnel with occupational exposure to horses was 19.5% [8]. In 2010, in the Netherlands, the veterinary staff was investigated during a MRSA outbreak at the university equine referral clinic and showed that 14.2% of personnel had a positive nasal colonization with MRSA [6].

Outside the equine hospital settings, the zoonotic risk appears to be limited. Belgian researchers investigated the MRSA prevalence of couples of non-hospitalized horses and their caretakers and showed a prevalence of 1.2% among 166 horses and 2.4% among their caretakers, indicating that healthy horses in this community pose a limited human health hazard [2].

In 2020 and 2022, MRSA was isolated from several surgical site infections (SSIs) in equine patients hospitalized in a Dutch equine referral clinic. Samples from the environment and staff members were obtained to investigate the possibility of the MRSA transmission among cases. MRSA isolates from horses, environment and staff from both time periods (2020 and 2022) were subjected to whole-genome sequencing to analyse the molecular characteristics and relatedness.

## Methods

### Cases

Bacteriological culture of nine cases (five in 2020 and four in 2022) of SSIs in an equine referral clinic tested MRSA positive. Five cases in 2020 occurred within a period of 3 weeks, and four cases in 2022 occurred in a period of 4 months. Eight of the nine horses had abdominal wound infections after colic surgery; one horse (E6) had a perivaginal abscess after a fetotomy. The horses with SSIs following colic surgery received an antibiotic treatment for 3–4 days with gentamicin 6.6 mg/kg intravenous (IV) and procaine penicillin G 20 000 IE/kg intramuscular (IM). Horse E6 was initially treated for 4 days with gentamicin 6.6 mg/kg IV and procaine penicillin G 20 000 IE/kg IM and subsequently for 3 days with trimethoprim-sulfadiazine 30 mg/kg per os (PO). All horses clinically recovered from their SSIs.

### MRSA isolation from the cases

Swabs from the SSIs were taken by the veterinarian after removing exudate from the wound and rinsing it with sterile saline. Bacteriological culture from wound swabs of the SSIs was performed through direct plating and culture at 37 °C on 5% sheep blood agar (Biotrading, Mijdrecht, the Netherlands). Presumptive isolates were identified as *S. aureus* by matrix-assisted laser desorption/ionization-time of flight (MALDI-TOF) Biotyper (Bruker, Delft, the Netherlands). Cefoxitin broth microdilution was used to screen for methicillin resistance, and quantitative real-time PCR (qPCR) was performed on cefoxitin-resistant strains to confirm the presence of the *mecA* gene [9].

### MRSA isolation from staff members and the environment

Additional samples were taken from contact staff members and the environment. Staff members took nasal swabs (self-sampling), and the environment was sampled by moving a swab over a surface of 3 cm^2^. Locations of the environmental screening were the preparation room, operating theatre, recovery stable, clipper machines, anaesthesia equipment, office, cabinets, x-ray room and the colic stable.

The investigation of staff members and environmental swabs was performed by both direct plating on Oxoid Brilliance MRSA 2 agar (Thermo Fisher Scientific, Basingstoke, UK) as well as selective enrichment using Mueller–Hinton Broth containing 6.5% NaCl (Biotrading, Mijdrecht, the Netherlands) and subculture after 24-h incubation on Oxoid Brilliance MRSA 2 agar. The identification was performed by MALDI-TOF Biotyper and qPCR for the confirmation of the *mecA* gene.

### Antimicrobial susceptibility testing

MICs were determined by broth microdilution using the commercially available automated MICRONAUT system (MERLIN Diagnostika GmbH, Bonn, Germany) using a customized panel for routine diagnostics at the Veterinary Microbiological Diagnostic Center. Clinical breakpoints of the Clinical and Laboratory Standards Institute [10] were used for interpretation.

### Whole-genome sequencing and genome analysis

Molecular characterization of the MRSA isolates was performed by Nanopore whole-genome sequencing. DNA was isolated using the DNeasy Ultra Clean Microbial kit (Qiagen, Venlo, the Netherlands). Nanopore sequencing was performed according to the protocol (RBK96.110) with flow cell type R9.4.1 on a MinION device (FLO-MIN106D) (Oxford Nanopore, Oxford, UK), using ‘super accurate’ basecalling. Reads were trimmed using Filtlong v0.2.1 and assembled using Flye v2.9.1 [11]. Assemblies were polished using Medaka and Homopolish. Prokka v1.14.5 was used to annotate the genomes, and a phylogenetic analysis was performed with parsnp v1.2 [12]. The parsnp core alignment was filtered for recombinations with gubbins v2.3.4 [13] and used to construct a minimum-spanning tree with GrapeTree [14]. Multilocus sequence typing was performed using MLSTFinder [15] and SCCmecFinder [16] for SCC*mec* typing. ResFinder and VirulenceFinder were accessed using cutoff values of >80% identity and >80% coverage (http://www.genomicepidemiology.org/). The basic local alignment tool (BLASTn) using the NCBI GenBank was applied for the confirmation of virulence genes.

## Results

### MRSA isolates from horses, staff members and environment

In 2020, MRSA was identified in two of the seven staff members (H1 and H2) and in none of the environmental swabs (*n*=5). In 2022, MRSA was identified in two of the eight staff members (H2 and H3) and in three of the eight environmental swabs. Staff member H2 was tested MRSA positive both in 2020 and 2022. In total, 16 MRSA isolates (9 equine, 3 environmental and 4 human) were long-read sequenced (Table S1, available in the online version of this article). In 2020, all isolates [five equine (E1–E5) and two human (H1 and H2)] belonged to ST398, *spa* type t11-SCC*mec* type Iva(2B). In 2022, four isolates [two equine (E6 and E9) and two human (H2 and H3)] belonged to ST398, *spa* type t6867-SCC*mec* Iva(2B), whereas the five remaining isolates [two equine (E7 and E8) and three environmental samples (S1–S3)] belonged to ST612, *spa* type t127-SCC*mec* IVd(2B). Staff member (H2) that tested positive in 2020 as well as in 2022 was colonized twice with MRSA ST398, however with distinct *spa* types [*spa* type t11-SCC*mec* type Iva(2B) in 2020 and *spa* type t6867- SCC*mec* Iva(2B) in 2022].

All ST398 isolates contained similar resistance genes and virulence genes (Table S1). These isolates contained resistance genes encoding for aminoglycoside resistance [*aac(6')-Ie/aph(2'')-Ia*], tetracycline resistance (*tetM*) and trimethoprim resistance (*dfrK*) and carried SCC*mec* type IV, like those described in clonal complex (CC) 398 lineages [17]. Phenotypical resistance was confirmed in these isolates for gentamicin and tetracycline, but not for the combination of trimethoprim-sulfamethoxazole, probably due to the absence of resistance genes for sulfonamides.

ST612 isolates harboured more resistance genes or chromosomal mutations encoding for resistance. They all contained macrolide resistance (*ermC*), aminoglycoside resistance [*aac(6')-Ie/aph(2'')-Ia*], tetracycline resistance (*tetM*) and trimethoprim resistance (*dfrC*) and mutations for sulfonamide resistance (*folP_ E208K* and *folP_F17L*), fluoroquinolone resistance (*gyrA S84L*) and rifampicin resistance (*rpoB H481N*). Phenotypical resistances were confirmed for all these antimicrobial classes.

In ST612 isolates, notable virulence genes were present, e.g. the human-specific immune evasion cluster (*scn, sak* and *sea*), additional enterotoxins (*seb*, *seq*) and the leukocidin genes *lukE* and *lukD* together with the *splA* and *splB genes* (Table S1). Furthermore, two plasmids were present with high sequence identity with pUSA500 [18] and pSWS371 [4,19].

### Phylogenetic analysis of MRSA isolates

**Fig. 1.**
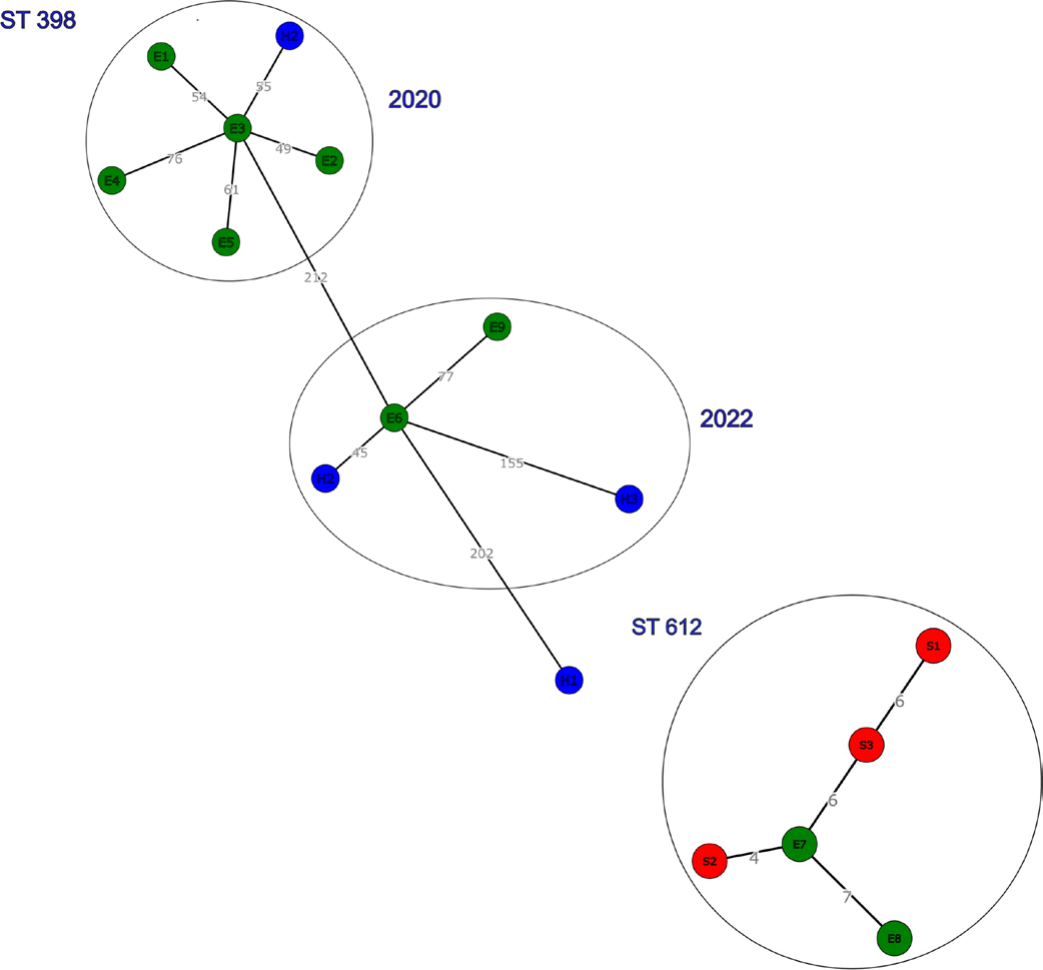
Minimum-spanning tree (MST) showing the core genome SNP distances of the MRSA ST398 cluster in 2020 and an ST398 and ST612 cluster in 2022. Sample types: E=equine (green), H=human (blue) and S=environment (red).

The ST398 isolates form three clusters (Fig. 1). Six out of seven ST398 isolates from 2020 (five equine and one human isolate) cluster together with an single nucleotide polymorphism (SNP) difference ranging from 49 to 76 SNPs (Fig. 1). The remaining human isolate from 2020 (H1) is likely unrelated to the other isolates of 2020 because of the SNP difference of 412 (Fig. 1). The third cluster is formed by four ST398 isolates from 2022 (two equine and two human isolates) with an SNP difference ranging from 45 to 155 SNPs (Fig. 1). Staff member H2 was involved in both ST398 clusters (2020 and 2022).

The ST612 isolates from horses and the environment (2022) showed very few SNP differences (ranging from 4 to 7 SNPs; Fig. 1) and share the same set of genes suggesting nosocomial transmission.

## Discussion

The molecular characterization of 16 MRSA isolates originating from 2 putative outbreaks of SSIs in 2020 and 2022 in an equine referral clinic showed that the isolates belonged to three different clusters. In 2020, ST398 was present in five SSIs and one staff member suggested nosocomial transmission. Even though another staff member (H1) also carried MRSA ST398, this isolate was not related and could therefore be considered as a separate introduction.

In 2022, nine isolates from four SSIs, two staff members and three environmental samples were divided into two distinct clusters, ST398 and ST612. It is likely that nosocomial transmission occurred within the clusters. ST398 was present in 2020 and 2022, but based on the SNP differences and other *spa* types, the ST398 isolates in both clusters were not related.

MRSA ST398 was present in seven of the nine SSIs in horses in this equine referral clinic. MRSA ST398 has been associated with the majority of SSIs caused by MRSA in horses in Europe and with the risk of persistence in the environment [20]. That one staff member (H2) MRSA ST398 carried both in 2020 and 2022, however with a different *spa* type, correlated with the observation that recurrent human infections with LA-MRSA CC398 can occur and reflect its low host specificity [21]. MRSA ST398 *spa* t011 and *spa* t6867 have been shown as frequent colonizers of staff working in equine clinics [21]. This ‘equine-associated’ MRSA, however, does play a minor role in human healthcare-associated infections and seems to cause limited dissemination outside veterinary hospitals [8]. Staff member H2 received a treatment for decolonization in 2020 from the physician; details on the treatment and follow-up of this person were not available. For this outbreak investigation, no additional healthcare information from staff members was obtained. Staff member H3 was colonized with a highly related ST398 isolate in 2022 but shows an SNP difference of 155 SNPs with the other ST398 isolates. This staff member was sampled almost 2 months after the equine sample E6 within this cluster, so this might have caused the rather large SNP difference. However, a separate introduction cannot be ruled out completely in this case.

To the best of our knowledge, MRSA ST612 has not been reported in Europe before, thus being an unexpected finding that MRSA ST612 caused an outbreak in this equine clinic in 2022. The closely related isolates from the environment and SSIs of the equine patients indicate a nosocomial transmission in 2022. The clinical presentation of the wound infection in the two horses with MRSA ST612 was not distinctive from the SSIs associated with MRSA ST398 infections. All horses recovered well. MRSA ST612 is widespread across South Africa associated with human infections and has been isolated from horses and incidentally in humans in Australia [4,22]. MRSA ST612 is a member of the CC8, which includes community-associated MRSA (CA-MRSA) USA300 and the closely related USA500 lineage. CA-MRSA USA300 and USA500 are reported in the USA, causing nosocomial infections in human hospitals [23], and are associated with the presence of the Panton–Valentine leukocidin (PVL) cytotoxin encoding genes *lukF-PV* and *lukS-PV*. However, in ST612, the *luk-ED* genes were present, which express low toxicity for human neutrophils [24]. In the ST398 isolates, no human- or equine-associated immune evasion genes were present [17], although, recently, a PVL-positive CC398 MRSA was detected in a Dutch hospital, underlining the importance of surveillance [25]. The MRSA isolates of both ST398 and ST612 in our study contained multiple virulence genes but lacked *lukF* and *lukS*.

The ST612 isolates contained two plasmids, pUSA500 and pSWS371, both carrying multiple transposable elements and resistance genes. The small plasmid pSWS371 has been described in MRSA CC398 isolates from broilers and carry *erm*(C) genes encoding for macrolide/lincosamide resistance [19]. The larger plasmid, pUSA500, has been described before in MRSA USA500 and carries *dfrC* responsible for trimethoprim resistance [18]. These plasmids can be transferred between bacteria which is a risk for the spread of antimicrobial resistance genes. Furthermore, the abundance of virulence and antimicrobial resistance genes present in the ST612 makes the treatment of these infections in horses challenging.

In all cases, the initial source and route of infection (and moment in time) could not be identified because the sampling of the environment and staff members took place after MRSA infection in the horses was confirmed. Hygienic measurements were immediately taken to reduce the risk of transmission within the clinic. Although analysing the transmission route was not the aim of this case study, monitoring of the animal hospital environment in the future could contribute to early detection and preventive hygienic measurements for clinical outbreaks [20]. This case report only describes a limited number of clinical MRSA ST612 isolates, but the finding of ST612 is worrying as it has an abundant set of virulence and resistance genes and is increasingly detected in infections in human patients [22,26]. The spread of epidemiologically successful MRSA lineages is driven by antimicrobial resistance [27], and surveillance will help us to understand the evolution, zoonotic potential and emergence of MRSA ST612.

## supplementary material

10.1099/jmm.0.001873 Uncited Table S1.

## Abbreviations

CA-MRSA: community-associated MRSA
CC: clonal complex
MALDI-TOF: matrix-assisted laser desorption/ionization-time of flight
MRSA: methicillin-resistant *Staphylococcus aureus*
MST: minimum-spanning tree
PVL: Panton–Valentine leukocidin
PVL: Panton–Valentine leukocidin
SNP: single nucleotide polymorphism
SSIs: surgical site infections
ST: sequence type
